# Toward a Global Perspective on Serrated Polyp Management

**DOI:** 10.1111/den.70294

**Published:** 2026-09-18

**Authors:** Joep E. G. IJspeert, Evelien Dekker

**Affiliations:** ^1^ Department of Gastrointestinal Oncology Netherlands Cancer Institute‐Antoni van Leeuwenhoek Amsterdam the Netherlands; ^2^ Department of Gastroenterology and Hepatology Amsterdam University Medical Center, Location VU Medical Center, University of Amsterdam Amsterdam the Netherlands; ^3^ Amsterdam Gastroenterology, Endocrinology & Metabolism Amsterdam the Netherlands; ^4^ Cancer Center Amsterdam Amsterdam University Medical Center Amsterdam the Netherlands

Despite growing recognition of the clinical significance of serrated polyps (SPs), substantial differences remain in how these lesions are approached across different regions of the world. With this in mind, Uraoka et al. provide an overview of current strategies for the management of SPs, highlighting key similarities and differences between Western and Japanese perspectives [1]. A notable strength of this review is its ambition to promote greater international harmonization. In doing so, it also makes clear that many recommendations are based on a still‐evolving evidence base and that several important questions remain unresolved. Framing these differences as a strict dichotomy between “the West” and Japan may oversimplify a more heterogeneous reality, as substantial variation also exists among the United States, the United Kingdom, and continental Europe. In this editorial, we aim to provide a European perspective on the discussed topics.

## 1 Histopathology

The nomenclature of SPs has been a source of considerable confusion for many years. In our view, greater international consistency is needed, and the current WHO classification provides the most appropriate framework for achieving this goal [2]. Accordingly, we favor the overarching term SP rather than serrated lesion. We also support the WHO approach of not distinguishing between low‐grade and high‐grade dysplasia within sessile serrated lesions (SSLs). Similarly, we do not consider superficially serrated adenoma (SuSA), as discussed in the review, to represent a distinct diagnostic entity. Based on its described histological and molecular characteristics, it appears to overlap substantially with the spectrum of traditional serrated adenomas (TSAs). The need for greater harmonization also extends to the histopathological diagnosis of SSLs. Uraoka et al. acknowledge important differences between Japanese diagnostic criteria and the current WHO classification. Such discrepancies may influence reported prevalence rates, risk estimates, and the comparability of studies across regions. To facilitate international consistency in both clinical practice and research, we believe that the WHO classification should serve as the common reference standard for the diagnosis and reporting of SSLs. Although most Japanese institutions have now adopted the current WHO classification, its transition has resulted in some clinical variability. In response, the Japan Serrated Lesion Expert Team (JSET) was established in 2023 and is currently developing a national expert consensus on clinical management.

## 2 Detection

Uraoka et al. discuss several strategies to improve the detection of SPs in the proximal colon, emphasizing the importance of a high‐quality colonoscopy. We fully agree that a high‐quality colonoscopy forms the cornerstone of adequate polyp detection. The ESGE has recently revised its Quality Improvement Initiative document, which is expected to be published shortly. The updated document advocates reporting quality indicators at the level of the individual endoscopist and recommends monitoring not only adenoma detection rate but also proximal SP detection rate (PSPDR). This recommendation is based on evidence suggesting that patients of endoscopists with high performance on both metrics have the lowest risk of postcolonoscopy colorectal cancer [3].

The authors further advocate the use of image‐enhanced endoscopy and a second‐pass of the proximal colon following application of an acetic acid–indigo carmine mixture (AIM). We greatly admire the meticulous and highly detailed approach of Japanese endoscopists for the detection and characterization of SPs. However, it remains uncertain whether an extensive second‐pass examination with AIM is feasible across a broad range of colonoscopy indications in routine clinical practice, particularly given the additional procedure time required. A more pragmatic opportunity for improvement may actually lie in monitoring PSPDRs and then targeted training of endoscopists with a low PSPDR [4]. Simply increasing awareness of the appearance and typical location of these lesions may be sufficient to improve detection. Patients in whom SSLs were detected at previous or during the current colonoscopy are at increased risk of having metachronous or synchronous SPs. In such cases, more meticulous inspection of the proximal colon seems warranted, and digital chromoendoscopy may serve as a valuable adjunct to enhance lesion detection and characterization.

## 3 Optical Diagnosis

One of the most striking global differences regarding the management of SSLs, highlighted by Uraoka et al., relates to the role of optical diagnosis of dysplasia. Whereas European guidelines generally advocate the removal of all lesions with an optical diagnosis of SSL, current Japanese guidelines recommend only resection of SSLs ≥ 10 mm in size or when dysplasia is suspected [1, 5]. This approach places considerable emphasis on the endoscopic size assessment of SSLs as well as on the ability of the endoscopist to accurately identify features suggestive of dysplasia. While conceptually attractive, we believe that this strategy is challenging in clinical practice. Regarding size, endoscopic assessment of polyp size is knowingly inaccurate. Second, endoscopic assessment of SSL size is even more challenging because the borders of a substantial proportion of SSLs are difficult to delineate. Last, recent literature demonstrated that the majority of SSLs with dysplasia are < 10 mm in size, calling into question the rationale for a 10‐mm threshold for resection [6]. Regarding optical diagnosis of dysplasia, robust prospective data demonstrating that endoscopists can reliably detect dysplasia within SSLs smaller than 10 mm and safely determine which lesions should be resected are, to our knowledge, lacking. It is plausible that dysplasia is most readily recognized when it presents as a distinct dysplastic nodule arising within an SSL, a feature that is often highlighted in the literature [7]. However, in our experience, this represents only a subset of SSLs with dysplasia. In many cases, the presence of dysplasia is considerably more subtle, making reliable endoscopic prediction in routine clinical practice challenging.

Given the above considerations, Uraoka et al. advocate resection whenever an SSL is suspected with high confidence, regardless of size or location [1]. To our knowledge, however, no studies have demonstrated that such a strategy is safe and feasible. Furthermore, there is insufficient evidence to support the assumption that larger hyperplastic polyps (HPs) and/or those located proximal to the rectosigmoid are invariably indolent. Finally, removal of these small lesions by cold snare polypectomy is safe and straightforward. Therefore, we favor a more pragmatic approach and recommend resection of any suspected SSL or TSA, irrespective of diagnostic confidence, as well as of HPs > 5 mm in the rectosigmoid and all HPs proximal to the rectosigmoid, regardless of size. It is noteworthy that the forthcoming JSET consensus statement is expected to recommend several pragmatic changes in clinical management that are more closely aligned with current European (ESGE) guidelines.

In our opinion, digital chromoendoscopy should be considered an essential component of the assessment of any polyp. It facilitates careful delineation and evaluation of potential dysplasia or invasive growth, thereby supporting optimal therapeutic decision‐making and complete resection. While dye‐based chromoendoscopy is regarded as an important adjunct for optical diagnosis in Japanese practice, its incremental value over high‐quality digital chromoendoscopy remains less clear. Given the additional time and procedural complexity involved, further evidence may be helpful to better define the situations in which dye‐based chromoendoscopy provides clinically meaningful benefits.

## 4 Resection

Another notable difference between Japanese and European practice is the preferred technique for SSL resection. In European practice, cold‐snare polypectomy, often combined with submucosal injection with blue dye for better delineation, has gained widespread acceptance and is considered the preferred approach for the removal of SSLs without suspected dysplasia [5]. For SSLs with dysplasia, meticulous evaluation to exclude invasive growth is advised, and conventional (diathermy‐based) en bloc endoscopic mucosal resection (EMR) is warranted. In contrast, Japanese practice places conventional EMR in a prominent role for SSL resection [1]. According to Uraoka et al., this reluctance for cold‐snaring appears to be driven largely by the limited availability of Japanese data evaluating the safety and efficacy of cold‐snare techniques for larger SSLs. However, we believe that the substantial international evidence supporting cold‐snare resection for SSLs warrants consideration and may justify the adoption of these techniques [5].

## 5 Surveillance

Interestingly, the more selective resection strategy advocated in Japan is accompanied by a relatively intensive surveillance policy. Current Japanese guidelines recommend surveillance colonoscopy after 3–5 years in patients with SSLs irrespective of lesion size [1] This contrasts with a more pragmatic strategy in Europe, where the focus is shifting toward performing a single high‐quality colonoscopy with complete removal of all clinically relevant lesions, not followed by surveillance for the majority of individuals [8]. The Japanese and European approaches may reflect fundamentally different philosophies: one emphasizing longitudinal surveillance of patients with SSLs, the other prioritizing complete eradication of all clinically relevant SPs during the index examination to minimize the need for future procedures. We believe that evidence‐based surveillance recommendations when suspected SSLs are left in situ are difficult to provide, as only limited data are available on the natural history and malignant potential of these lesions. In contrast, important evidence is emerging for strategies of resecting all clinically relevant SPs. For instance, the ongoing EPOS III trial will report soon on the safety of extending surveillance intervals to 5 years after resection of clinically relevant SPs and, importantly, will provide data on the absolute risk of colorectal cancer to guide future surveillance recommendations [9].

Finally, the authors recommend annual surveillance for patients with serrated polyposis syndrome (SPS). While annual surveillance may be appropriate for selected high‐risk individuals, a uniform 1‐year interval may not be necessary for all patients with SPS. When colonoscopies are performed at a consistently high level of quality, including complete removal of all clinically relevant lesions, surveillance intervals can be safely extended in a substantial proportion of patients [10].

In conclusion, although we share the authors' ambition to optimize the detection and management of SPs, we would place a different emphasis on several key aspects of clinical management. In our view, prioritizing quality monitoring of colonoscopy, targeted endoscopist training, complete resection of all (suspected) clinically relevant SPs, and evidence‐based surveillance may provide a more pragmatic approach for clinical practice.

## Author Contributions

Original draft: E.D., J.E.G.I. Critical revision of the manuscript: E.D., J.E.G.I.

## Funding

The authors have nothing to report.

## Conflicts of Interest

E.D.: Honoraria for consultancy from Olympus, Fujifilm, Norgine, Exact Sciences, and ProMedCS. Speakers' fees from Olympus, Norgine, IPSEN/Mayoly, FujiFilm, Steris, and Pentax. Endoscopic equipment on loan from FujiFilm. J.E.G.I.: no conflicts of interest.

## Linked Articles

This article is linked with Uraoka et al. To view this article, visit https://doi.org/10.1111/den.70197.

## Data Availability

Data sharing not applicable to this article as no datasets were generated or analysed during the current study.
